# Entropy Production in an Electro-Membrane Process at Underlimiting Currents—Influence of Temperature

**DOI:** 10.3390/e27010003

**Published:** 2024-12-25

**Authors:** Juan Carlos Maroto, Sagrario Muñoz, Vicenta María Barragán

**Affiliations:** 1 Department of Electronics, Automation, and Communications, Comillas Pontifical University, 28049 Madrid, Spain; jcmaroto@comillas.edu; 2 Department of Science and Aerospace, Universidad Europea de Madrid, 28670 Madrid, Spain; 3 Department of Structure of Matter, Thermal Physics and Electronics, Faculty of Physics, Complutense University of Madrid, 28040 Madrid, Spain; smsm@ucm.es

**Keywords:** cation-exchange membrane, limiting current, diffusion boundary layers, concentration polarization, entropy production, current–voltage curve, saline concentration gradient

## Abstract

The entropy production in the polarization phenomena occurring in the underlimiting regime, when an electric current circulates through a single cation-exchange membrane system, has been investigated in the 3–40 °C temperature range. From the analysis of the current–voltage curves and considering the electro-membrane system as a unidimensional heterogeneous system, the total entropy generation in the system has been estimated from the contribution of each part of the system. Classical polarization theory and the irreversible thermodynamics approach have been used to determine the total electric potential drop and the entropy generation, respectively, associated with the different transport mechanisms in each part of the system. The results show that part of the electric power input is dissipated as heat due to both electric migration and diffusion ion transports, while another part is converted into chemical energy stored in the saline concentration gradient. Considering the electro-membrane process as an energy conversion process, an efficiency has been defined as the ratio between stored power and electric power input. This efficiency increases as both applied electric current and temperature increase.

## 1. Introduction

Concentration polarization at the surface of an ion-exchange membrane arises from the difference between the transport numbers of an ionic species in the membrane and in the free solution. At equilibrium, without electric current or other driving forces, the bulk concentration is uniform throughout the external solution, including the diffusion boundary layers. When an electrical current is applied, ions start to migrate to and through the ion exchange membrane. Since the electrical current is carried by both cations and anions in free solutions, but only by counterions through the ion-exchange membrane, the concentration profile across the boundary layer gives rise to a diffusional flux of ions to the membrane surface to balance the unequal migration fluxes; that is, polarization occurs. This effect is very important in applications involving ion-exchange membranes in electrolyte media, such as electrodialysis [1]. It is an essential limiting factor in the performance of electrically driven processes in general. Therefore, these phenomena have been widely studied in the literature [1,2,3,4,5,6,7,8,9,10,11,12,13,14,15,16,17,18,19,20].

Usually, polarization phenomena occurring with ion exchange membranes are studied using models based on the Nernst–Planck transport equation [3,4,5,7,9,10,11,12,14,15,17,18,19,20]. More recent works mainly focus on phenomena associated with overlimiting transport [4,7,11,13,14,15,20].

However, studies examining these systems from a thermodynamics point of view are rarer. Chehayeb and Lienhard [21] analyzed the entropy generation in electrodialysis, only considering the electric driving force. Generous et al. [22] also studied entropy generation of electrodialysis desalination using multi-component solutions. Ślezak et al. [6] used the non-equilibrium thermodynamics-based Kedem–Katchalsky equations to describe the influence of the concentration boundary layer on membrane potential in a single membrane system. Ślezak et al. [23] used the same formalism to evaluate S-entropy production in a single membrane system in concentration polarization conditions. However, in both cases, only a concentration gradient was initially imposed in the system.

Non-equilibrium thermodynamics gives a unified method to study coupled transport processes [24]. Kjelstrup and Bedeaux [25] have encouraged the use of the non-equilibrium thermodynamics formalisms to describe transport in heterogeneous media. It can describe coupled transport processes, such as those occurring under concentration polarization conditions. Entropy production in a system can be used to assess how energy resources are exploited within the system. Non-equilibrium thermodynamics formalism has been applied to study fuel cells [26], thermoelectric cells [27], or reverse electrodialysis processes [28]. The work of Manganelly et al. [29] showed that entropy production minimization can be used as a method to optimize the use of energy resources in a system.

However, in most cases, the formalism of the non-equilibrium thermodynamics is used to determine relationships between flows and forces present in the system rather than as a diagnostic tool for analyzing energy system.

The general purpose of this work is to analyze entropy production in an electrically driven process in a single membrane system under polarization concentration conditions in the underlimiting regime, considering the system as a heterogeneous medium. To this end, we combine classical polarization theory and irreversible thermodynamics formalism. The objective is to identify the different irreversible processes that contribute to entropy production in the system and evaluate the parameters that influence it.

## 2. Fundamentals

### 2.1. The System

The thermodynamic system considered is a heterogeneous system formed by different subsystems: electrodes, bulk solution, concentration boundary layers, and membrane.

**Figure 1:**
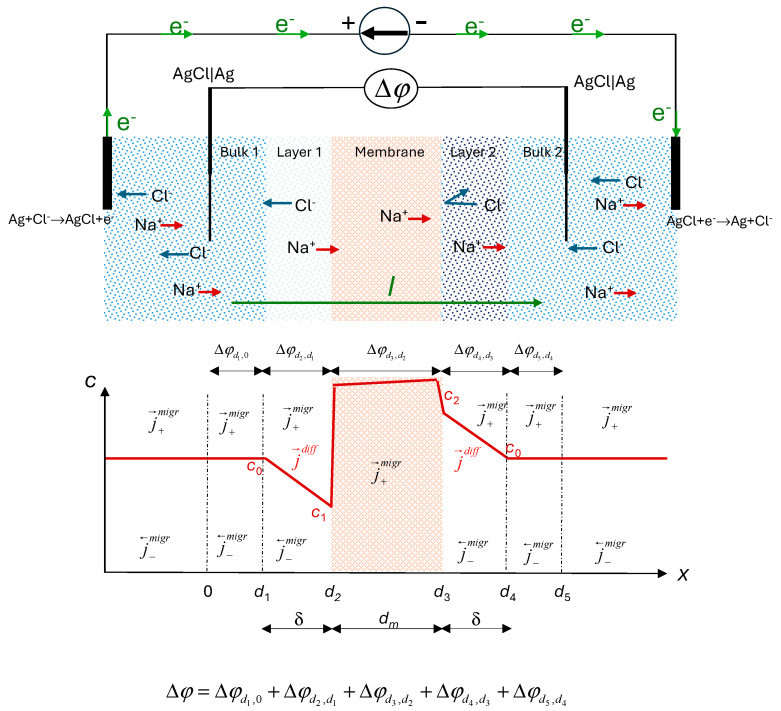
Sketch of the ion-exchange membrane system under study: An electric current passes through a cation exchange membrane separating two aqueous solutions of NaCl of equal concentration *c*_0_ (**above**). The Ag|AgCl electrodes are reversible to Cl^−^ ion concentration profiles and different fluxes across the system (**bottom**).

Figure 1 (above) illustrates the system under study. It consists of a cation-exchange membrane separating two identical 1:1 electrolyte solutions of bulk concentration *c*_0_ maintained at the same temperature and hydrostatic pressure. In this configuration, a constant electric current passes through the membrane system. As it is well known, due to the different mobilities of the counterions in membrane and in free solution phases, a current-induced concentration gradient is established within the solution and perpendicular to the membrane surface. The Nernst films model [30] is often used to analyze this phenomenon. This model assumes the formation of polarization layers adjoining the membrane at the two surfaces. Within these layers, termed diffusion boundary layers, convection is absent, and mass transfer is governed by diffusion and migration, resulting in a linear concentration profile (Figure 1, bottom) under steady-state conditions. The Nernst model overlooks the impact of convection on transport. Levich [31] refined this model, demonstrating that convective transport within the diffusion layers results in a smooth, monotonic concentration profile that asymptotically approaches the bulk concentration. For this study, we adopt the Nernst model for simplicity. Linear concentration profiles are obtained in this model. Due to the small concentration gradient existing in the system, a significant convection effect is not expected.

This phenomenon, termed polarization concentration, significantly affects transport properties, particularly the cell voltage drop, Δ*φ*, between bathing solutions on either side of the membrane.

The transport processes considered occur along the horizontal axis of the cell, referred to as the *x*-axis. Fluxes through the membrane are treated as scalar components of the vectorial flux in this direction. The membrane surfaces can be assumed to be in local equilibrium. The electric current is applied via reversible Ag|AgCl electrodes, and we also use reversible Ag|AgCl electrodes for measuring the electric potential difference. Treating the system as a multi-layer system, the total voltage drop comprises contributions of the different parts of the system: external electric circuit, electrodes, bulk solution, boundary solution layers, and membrane.

In the bulk solution, far from the diffusion boundary layers, ion transport occurs solely by migration, as there are no concentration gradients perpendicular to the membrane surface.

Within the highly permselective cation-exchange membrane, due to the anion exclusion, there is no significant concentration gradient inside it. Anions are more or less completely excluded from the membrane, and their flux by migration and diffusion in opposite directions is compared to that of the cations, which is generally small. Thus, it can be assumed, to a first approximation, that the transport of cations through the highly permselective membrane occurs primarily via migration driven by the electrical potential gradient [32].

In the diffusion boundary layers, ions are transported through a combination of migration and diffusion mechanisms (Figure 1, bottom). Reversible electrodes in the system are maintained under identical conditions of concentration, temperature, and hydrostatic pressure.

### 2.2. The Current–Voltage Curve of a Single Membrane System

**Figure 2:**
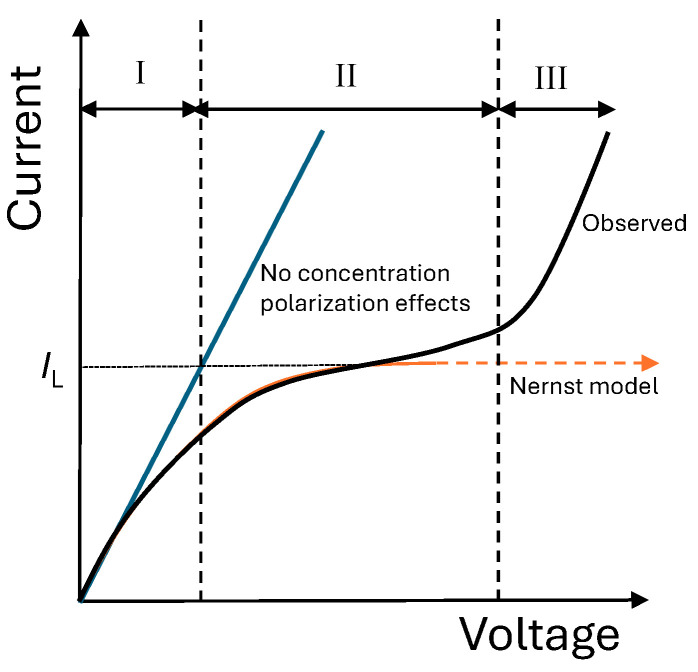
Sketch of a typical current–voltage curve of an ion-exchange membrane in an electrolyte solution (in black), showing the three distinct regions: (I) linear increase, (II) plateau, and (III) overlimiting transport. Profile predicted by the Nernst model (in red) and linear voltage–current curve (in blue) for a system without concentration polarization effects are also shown.

According to the classical theory of concentration polarization [30], the electric current, *I*, should increase linearly with voltage, Δ*φ*, at low voltages and then rise more slowly, eventually reaching a limiting value. This limiting value, termed limiting current, *I_L_*, would correspond to the value of the current at which the concentration of the solution becomes zero at the membrane surface. At this stage, diffusion is unable to supply enough ions to compensate for the migration flux through the membrane, resulting in an ion depletion at this membrane surface. In a real system, however, the current–voltage curve exhibits a characteristic shape with three regions (Figure 2) clearly distinguished. After the linear relationship (I), the curve reaches a region in which the current remains nearly constant despite increasing voltage, a region known as the plateau region (II). Finally, a new region (III) of current increasing with voltage is observed.

It indicates that the concentration of counterions does not become zero in the membrane surface due to the activation of overlimiting transport mechanisms, such as dissociation of water, gravitational convection, and electroconvection, which allow the increase in ionic transfer through the membrane [11,14]. Our study focuses on the underlimiting regime at currents below the limiting value.

Using the classical theory of concentration polarization, the Peers’ equation [32] is obtained, which allows us to estimate the value of the limiting current, *I_L_*, if the solute concentration, *c*_0_, the diffusion coefficient of solute in water, *D*, the boundary layer thickness, *δ*, and the counter-ion transport numbers in the membrane, ${\bar{t}}_{+}$, and in free solution, $t_{+}$, are known:

(1) $$I_{L}=\frac{AFDc_{0}}{\delta \left({\bar{t}}_{+}-t_{+}\right)}$$

where *F* is the Faraday constant and *A* is the membrane effective area. This equation is obtained by considering Nernst’s linear concentration profile under steady-state conditions, where the concentration of the solution becomes zero at the membrane surface and the limiting current is achieved. The boundary layer thickness also depends on the hydrodynamic conditions.

For currents *I* < *I_L_*, the concentrations at the dilute (*c*_1_) and concentrated (*c*_2_) solutions in the membrane/solution interfaces can be expressed as follows:

(2) $$\begin{array}{l} c_{1}=c_{0}\left(1-\frac{I}{I_{L}}\right)\\ c_{2}=c_{0}\left(1+\frac{I}{I_{L}}\right) \end{array}$$

In this approach, an equation can be derived for the electric potential difference $\Delta \varphi_{AB}$ between two fixed points placed on both sides of the membrane in the corresponding bulk solution when the stationary state has been reached after the injection of an electric current in the membrane system. This equation, obtained in a previous work [5], is expressed as follows:

(3) $$\Delta \varphi_{AB}=\left(R_{0}-\frac{RT}{F\Delta t_{+}I_{L}}\right)I+\frac{RT}{F}\left[\left(2\Delta t_{+}\right)+{\left(2\Delta t_{+}\right)}^{-1}\right]\ln\left(\frac{1+\frac{I}{I_{L}}}{1-\frac{I}{I_{L}}}\right)$$

where *T* is the absolute temperature, *R* is the gas constant, $\Delta t_{+}=\left({\bar{t}}_{+}-t_{+}\right)$ and *R*_0_ indicate the total ohmic resistance of the system before the polarization layers are formed. This expression has two contributions, one due to the polarization potential established through the membrane system due to the concentration gradient and the other due to the change in the ohmic resistance of the system as a consequence of the formation of the layer.

Equation (3) is only applicable to electric currents such as *I* < *I_L_*. It predicts that the electric potential difference tends to infinity as *I* becomes *I_L_* and the polarization potential as well as the resistance of the depletion layer tend to infinity in the Nernst model.

### 2.3. The Non-Equilibrium Thermodynamics Formalism

The system in Figure 1 can be analyzed with the non-equilibrium thermodynamic formalism. As an isothermal electrochemical cell, it exhibits coupled transport of mass and charge. It consists of different parts that will contribute to the total entropy production. In the considered system, the only external force applied is an electric potential gradient.

According to the irreversible thermodynamics approach, for a homogeneous phase, the local entropy production, σ, which indicates the change in the entropy in a volume element, can be expressed as a function of the fluxes, *J_i_*, and forced, *X_i_*, existing in the system as:

(4) $$\sigma =\sum_{i=1}^{n}J_{i}X_{i}$$

where *n* is the number of independent fluxes. Irreversible thermodynamics assumes linear relationships between fluxes and forces, and, in general:

(5) $$J_{i}=\sum_{j=1}^{n}L_{ij}X_{j}\;\mathrm{for}\;j\;=\;1,\;\ldots ,\;n$$

where *L_ij_* are the linear phenomenological coefficients. It is assumed that the linear relations are locally valid.

The total entropy production due to the irreversible processes occurring in the system, $\frac{dS_{i}}{dt}$, is obtained by integrating Equation (4) over the entire volume *V* of the system considered:

(6) $$\frac{dS_{i}}{dt}=\int_{V}\sigma dV$$

Considering one-dimensional transport in the normal direction (*x*), the entropy production for the whole path, with a cross-section *A*, Equation (6) can be written as follows:

(7) $$\frac{dS_{i}}{dt}=A\int \sigma \left(x\right)dx$$

The wasted or lost work per unit time is the energy dissipated as heat in the surrounding area and, in the isothermal system, can be expressed as [26]:

(8) $$W_{dissipated}=T\frac{dS_{i}}{dt}$$

Systems with membranes are heterogeneous systems, consisting of bulk and surface parts, all of them with entropy production: electrode surfaces, bulk solution, polarization layers, and membranes. The total entropy production between two fixed points placed at both sides of the membrane in the corresponding bulk solution when the stationary state is reached can be calculated by applying Equation (7) to the different homogeneous parts of the system placed between these two points.

In our isothermal and isobaric system, only one force is externally applied, the electric potential gradient. However, as previously described, the polarization concentration effects caused by the pass of the electric current will result in a concentration gradient, and coupled transports of mass and charge will also occur in the cell.

The local entropy production is expressed as follows:

(9) $$\sigma =\sum_{j}J_{j}\left(-\frac{1}{T}\frac{\partial \mu_{j,T}}{\partial x}\right)+J\left(-\frac{1}{T}\frac{\partial \varphi }{\partial x}\right)+\sum_{r}r\left(-\frac{\Delta_{r}G}{T}\right)$$

where *J_i_* and *J* are, respectively, mass and electric charge flux densities, *μ_j_*_,*T*_ is the chemical potential at isothermal conditions of the *j* component, and *r* and Δ*_r_G* are, respectively, the velocity and the reaction Gibbs energy of reaction *r*. If there are no reactions present in the system, the last term in Equation (9) is null.

Coupling between mass and electric charge fluxes can occur in the system. According to Equation (5), if there is only one reaction occurring in the system, the flux equations can be written as follows:

(10) $$\begin{array}{l} J_{i}=\sum_{j}L_{ij}\left(-\frac{1}{T}\frac{\partial \mu_{j,T}}{\partial x}\right)+L_{i\varphi }\left(-\frac{1}{T}\frac{\partial \varphi }{\partial x}\right)\\ J=\sum_{j}L_{\varphi j}\left(-\frac{1}{T}\frac{\partial \mu_{j,T}}{\partial x}\right)+L_{\varphi \varphi }\left(-\frac{1}{T}\frac{\partial \varphi }{\partial x}\right)\\ r=L_{rr}\left(-\frac{1}{T}\Delta_{r}G\right) \end{array}$$

In the studied case, connecting leads and reversible electrodes are at the same temperature, pressure, and concentration. The only reaction presented in the system is the reversible reaction corresponding to the electrode surfaces required to change the charge carrier from chloride in solution to an electron in the external circuit. Thus, the main contribution to the entropy production will be due to the electrolyte solution, including boundary diffusion layers, and the membrane.

In analyzing membrane processes, it is more convenient to transform the previous equation into an expression involving the flux of neutral salt, *J_s_*, more than the individual ion fluxes [33]. We choose the membrane as the natural frame of reference for the fluxes. Neglecting the water flux, we have two fluxes (salt and charge) and two involved forces (electric and chemical potential gradients)—Equation (9) can be written as a function of the salt flux as:

(11) $$\sigma =-J_{s}\frac{1}{T}\frac{\partial \mu }{\partial x}-J\frac{1}{T}\frac{\partial \varphi }{\partial x}$$

and Equation (10) as:

(12) $$\begin{array}{l} J_{s}=-L_{\mu \mu }\frac{1}{T}\frac{\partial \mu }{\partial x}-L_{\mu \varphi }\frac{1}{T}\frac{\partial \varphi }{\partial x}\\ J=-L_{\varphi \mu }\frac{1}{T}\frac{\partial \mu }{\partial x}-L_{\varphi \varphi }\frac{1}{T}\frac{\partial \varphi }{\partial x} \end{array}$$

where we have dropped subscript *T* in the chemical potential gradient since the system is isothermal.

Introducing the salt transference coefficient, *t_s_*, defined as the ratio of the salt flux and the electric current density at uniform composition, and the salt diffusion coefficient from Fick’s law at zero current, *D*, the second expression in Equation (12) allows us to obtain a relation between both fluxes:

(13) $$J_{s}=-D\frac{\partial c}{\partial x}+\frac{t_{s}}{F}J$$

where the relation:

(14) $$\mu_{NaCl}=\mu_{NaCl}^{0}+2RT\ln c_{NaCl}$$

has been used for relating the salt chemical potential and concentration; mean activities of ions of value 1 are considered. For Ag|AgCl electrodes reversible to the ion Cl^−^, the salt transference number is equal to the transport number of the cation *t*_+_, with:

(15) $$t_{+}+t_{-}=1$$

where *t*_−_ is the anion transport number.

## 3. Materials and Methods

The membrane used in this work was a commercial Nafion 117 membrane, a homogenous, highly selective cation-exchange membrane manufactured by Dupont Inc. According to the data provided by the manufacturer, this membrane has a nominal thickness of 183 μm and an IEC of 0.94 meq/g.

Sodium chloride (pro-analysis grade) and deionized, doubly distilled water were used to prepare the electrolyte solutions.

**Figure 3:**
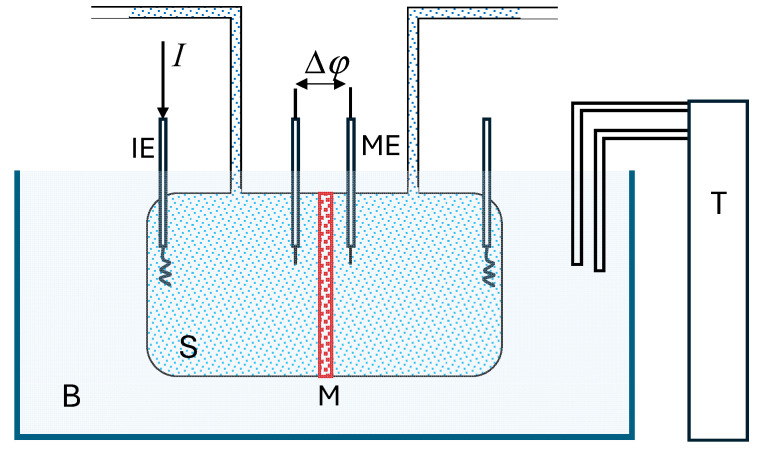
Sketch of the experimental setup for measuring current–voltage curves. I: electric current; B: bath; T: thermostat; S: solution; M: membrane; IE: Ag|AgCl injecting electrode; ME: voltage Ag|AgCl electrode.

A sketch of the experimental setup used for measuring current–voltage curves, already described elsewhere [5], is shown in Figure 3. The membrane was vertically placed, separating two glass chambers containing two NaCl solutions of the same concentration (5 molm^−3^). The exposed membrane surface area was 9.04 × 10^−4^ m^2^. Both chambers were maintained at the same pressure, and all the experiments were carried out under isothermal conditions by immersing the cell in a thermostatic bath.

The solutions in the chambers were under natural convection conditions, without any stirring. A constant electric current was applied by means of a large active surface Ag|AgCl electrode, and Ag|AgCl probe electrodes were positioned on either side of the membrane to record the electric potential difference between them.

Current–voltage curves were obtained by measuring the electric potential difference at varying current levels. This process was repeated at different temperatures ranging from 3 to 40 °C.

At each temperature, the electric conductivity of the solution was measured with a conductivity meter, JENCO Model 1671. Closed vessels containing the solution were immersed in a thermal bath at the selected temperature. Once the thermal equilibrium was reached, the conductivity of the solutions was measured using a suitable conductivity probe. The accuracy of the conductivity measurements was 1 μScm^−1^.

## 4. Results and Discussion

### 4.1. Properties of the Electrolyte Solution

The electrical conductivity, *κ*, of the electrolyte solution has been measured at each temperature. The results are shown in Table 1. From these values, the diffusion coefficient, *D*, at each temperature was calculated using the following relationship [34]:

(16) $$D=\frac{\Lambda RT}{2F^{2}}$$

where *Λ* is the specific conductance, defined by the following expression:

(17) $$\Lambda \left({\mathrm{Scm}}^{2}{\mathrm{mol}}^{-1}\right)=100\frac{\kappa \left({\mathrm{Scm}}^{-1}\right)}{c(M)}$$

and *c* is the molar concentration of the electrolyte. The results obtained are also shown in Table 1. Transport numbers of Na^+^ at different temperatures have been estimated using information from literature [35,36].

The data in Table 1 indicate that increasing temperature enhances the transport properties of the solution phase.

### 4.2. Current–Voltage Curves

**Figure 4:**
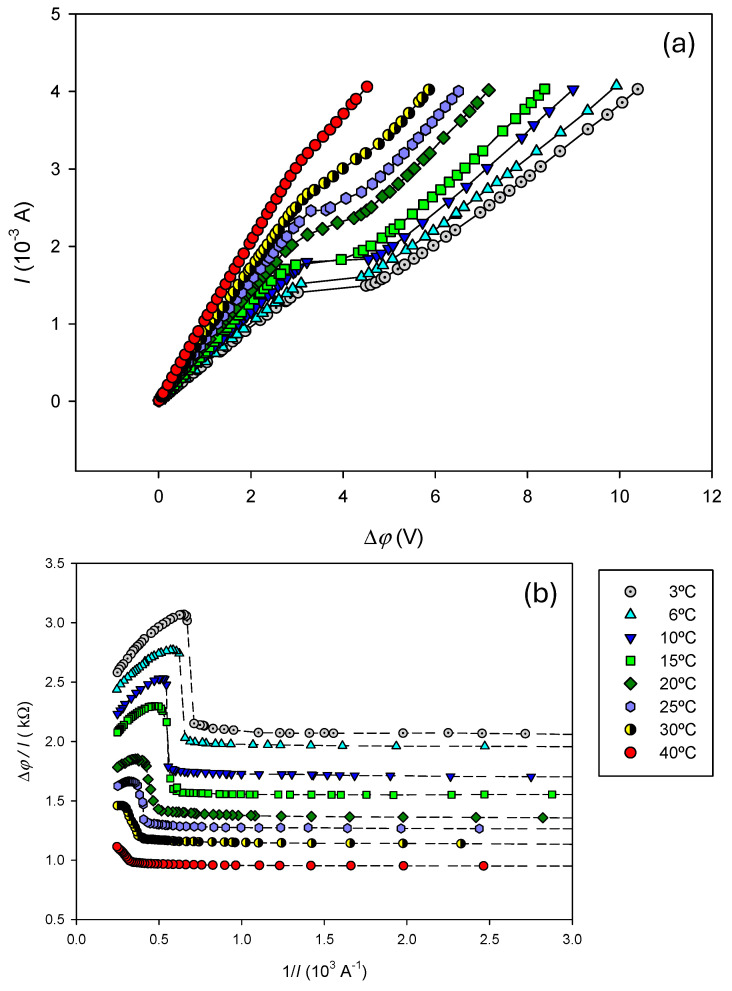
(**a**) Current–Voltage curves and (**b**) corresponding Cowan plots at different temperatures. Dashed lines are included as visual guides.

Current–voltage curves were measured at different temperatures. They are presented in Figure 4a.

At temperatures below 30 °C, the obtained curves exhibit the typical profile for cation-exchange membranes immersed in electrolyte solutions, showing the three usual characteristic regions described earlier. Visual inspection of Figure 4a reveals that the second region, corresponding to the plateau, occurs at greater current and becomes less pronounced as temperature increases. Moreover, for 30 and 40 °C, a defined plateau is no longer clearly appreciated.

In the linear region (I) and in the approximately linear segment in region (III), the trend observed with increasing temperature may be attributed to a reduction in the system’s electric resistance. The electric conductivity of an electrolyte solution increases with temperature [34,35,36], as do the ion transport number and the salt diffusion coefficient, facilitating ion transport. The electric conductivity of Nafion 117 is also temperature dependent. Results from the literature have shown that the electric conductivity of the H^+^-form Nafion membrane is not a monotonic function of the temperature. Initially it increases with temperature, reaches a maximum value of about 60 °C, and then decreases [37]. In Na^+^-form, an Arrhenius relationship has been observed for the electric conductivity with temperature [38].

In region II, the plateau width decreases with increasing temperature, and it becomes less defined, eventually disappearing entirely at 25 and 30 °C. At these temperatures, what is observed is rather an inflection on the current voltage curve. As the temperature increase facilitates ion transport in solution, it may indicate that cation concentration drop on the diluted surface of the membrane decreases with temperature, reducing the voltage drop, also affecting the different mechanisms involved in the overlimiting regime. Further studies are underway to better understand this behavior.

#### 4.2.1. Determination of the Limiting Current Value

The limiting current *I_L_* at each temperature was determined from the corresponding Cowan plots, presented in Figure 4b. The results are presented in Table 2. As was expected from the observation of plateaus in Figure 4a, *I_L_* increases with temperature.

#### 4.2.2. Determination of R_0_ and Counterion Transport Number in the Membrane ${\bar{t}}_{+}$

Using the measured limiting currents, the experimental data (Δ*φ*, *I*) in region I were fitted to Equation (3) using a minimization χ^2^ method. It allowed us to estimate parameters *R*_0_ and ${\bar{t}}_{+}$ at each temperature. Results are shown in Table 2. As expected, *R*_0_ decreases with increasing temperature, likely due to the enhanced electrical conductivity of the electrolyte. As the electrical conductivity of Nafion membranes is high [39], the greater contribution to *R*_0_ must be due to the electrolyte contribution. The results indicate that the Na^+^ transport number in the membrane hardly varies with temperature.

By utilizing data from Table 1 and Table 2, the thickness of the boundary diffusion layers at each temperature was estimated using the Peer’s equation. Results are also shown in Table 2. The values obtained agree with typical values given in the literature for similar systems [8,10]. A slight increase in layer thickness was observed for temperatures above 20 °C. Experimental observations have confirmed that the Nernst model, which reasonably coincides with the theoretical diffusion boundary layer thickness calculated from the limiting current, effectively depicts the transport phenomena in ion-exchange membrane systems. By measuring the potential drop with a mobile micro-electrode at various distances from a cation-exchange membrane [40], it was observed that the diffusion boundary layers existed in the range of 300–350 µm. Thicknesses of a similar order of magnitude to those found in this work have been observed using laser interferometry [41].

### 4.3. Entropy Production

We apply Equations (7) and (11) to estimate the entropy production in each part of the system. The total entropy production is obtained by the following expression:

(18) $$\frac{dS_{i}}{dt}=A\int_{\mathrm{bulk}}\sigma \left(x\right)dx+A\int_{\mathrm{layers}}\sigma \left(x\right)dx+A\int_{\mathrm{membrane}}\sigma \left(x\right)dxA$$

In the bulk solutions, where no chemical potential gradient exists, ions move solely by migration. From Equation (11), the dissipation function, representing the local energy dissipated as heat due to irreversible processes, is expressed as:

(19) $$T\sigma =-J\frac{d\varphi }{dx}$$

From Equation (7), and assuming steady-state conditions with constant fluxes, the entropy production in the bulk solution is given by:

(20) $$T{\left.\frac{dS_{i}}{dt}\right|}_{\mathrm{bulk}}=A\int T\sigma \left(x\right)dx=-AJ\Delta \varphi_{\mathrm{bulk}}$$

According to Figure 1 and the second expression in Equation (12), the electric potential difference across the bulk phase is given by:

(21) $$\Delta \varphi_{bulk}=\Delta \varphi_{d_{1},0}+\Delta \varphi_{d_{5},d_{4}}=-J\frac{d_{\mathrm{bulk}}T}{L_{\varphi \varphi }}$$

where *d*_bulk_ is the thickness of the two bulk phases in the cell. Considering the electric conductivity for a uniform distribution of salt,

(22) $$\kappa \equiv \frac{L_{\varphi \varphi }}{T}$$

the total heat dissipation in the bulk solution can be expressed in terms of the total electric resistance in the bulk phase, *R*_bulk_, and the electric current circulating along the system:

(23) $$T{\left.\frac{dS_{i}}{dt}\right|}_{\mathrm{bulk}}=\frac{AJ^{2}d_{\mathrm{bulk}}}{\kappa_{\mathrm{bulk}}}=I^{2}R_{\mathrm{bulk}}$$

where *I* = *J.A.* In the boundary diffusion layers, ions are transported by both migration and diffusion. Consequently, both terms in Equation (11) contribute to the entropy production. Using Equations (13) and (14), the total heat dissipation in layers 1 and 2 can be expressed as follows:

(24) $$\begin{array}{l} T{\left.\frac{dS_{i}}{dt}\right|}_{\mathrm{layer}1}=2ARTD\left(\frac{c_{1}-c_{0}}{\delta }\right)\ln\frac{c_{1}}{c_{0}}-AJ\Delta \varphi_{d_{2},d_{1}}-\frac{AJRT2t_{+}}{F}ln\frac{c_{1}}{c_{0}}\\ T{\left.\frac{dS_{i}}{dt}\right|}_{\mathrm{layer}2}=2ARTD\left(\frac{c_{0}-c_{2}}{\delta }\right)\ln\frac{c_{0}}{c_{2}}-AJ\Delta \varphi_{d_{4},d_{3}}-\frac{AJRT2t_{+}}{F}ln\frac{c_{0}}{c_{2}} \end{array}$$

where the electric potential difference in the boundary diffusion layers, $\Delta \varphi_{d_{2},d_{1}}$ and $\Delta \varphi_{d_{4},d_{3}}$ can be written as [16,33]:

(25) $$\begin{array}{l} \Delta \varphi_{d_{2},d_{1}}=-\frac{J\delta }{\kappa_{l_{1}}}-\left(1-2t_{+}\right)\frac{RT}{F}ln\frac{c_{1}}{c_{0}}\\ \Delta \varphi_{d_{4},d_{3}}=-\frac{J\delta }{\kappa_{l_{2}}}-\left(1-2t_{+}\right)\frac{RT}{F}ln\frac{c_{0}}{c_{2}} \end{array}$$

where $\kappa_{l_{1}}$ and $\kappa_{l_{2}}$ are the electric conductivity of dilute and concentrate layers, respectively, and the same thickness has been considered for both layers. By combining Equations (24), (25) and (22) for layer conductivity, the total heat dissipation in both layers can be written as follows:

(26) $$T{\left.\frac{dS_{i}}{dt}\right|}_{\mathrm{layers}}=I^{2}R_{layers}+2ARTD\left(\frac{c_{0}I}{\delta I_{L}}\right)\ln\frac{c_{2}}{c_{1}}-I\left(1-4t_{+}\right)\frac{RT}{F}ln\frac{c_{2}}{c_{1}}$$

where *R_layers_* indicates the ohmic resistance of both layers.

Entropy production within the membrane was evaluated considering its high selectivity. In this case, cations move predominantly due to migration under the influence of the electric potential gradient. Neglecting water flux, the expression for estimating the heat dissipation inside the membrane is similar to Equation (19) used for determining the bulk solution contribution:

(27) $$T{\left.\frac{dS_{i}}{dt}\right|}_{membrane}=A\int T\sigma \left(x\right)dx=-AJ\Delta \varphi_{membrane}=-AJ\Delta \varphi_{d_{3,}d_{2}}$$

but in this case, there are two contributions to the electric potential difference: one is the ohmic voltage drop across the membrane, and the other is the membrane potential [30]. Thus,

(28) $$\Delta \varphi_{d_{3},d_{2}}=-\frac{Jd_{m}}{\kappa_{m}}-\left(1-2{\bar{t}}_{+}\right)\frac{RT}{F}ln\frac{c_{2}}{c_{1}}$$

where *κ_m_* is the membrane electric conductivity. Combining Equations (27) and (28) and using Equation (22) for the membrane conductivity, the total heat dissipation within the membrane is given by:

(29) $$T{\left.\frac{dS_{i}}{dt}\right|}_{membrane}=I^{2}R_{m}+I\left(1-2{\bar{t}}_{+}\right)\frac{RT}{F}ln\frac{c_{2}}{c_{1}}$$

The total heat dissipation is obtained by adding all contributions indicated in Equations (23), (26), and (29), resulting in:

(30) $$T{\left.\frac{dS_{i}}{dt}\right|}_{total}=I^{2}R_{total}+I\frac{2t_{+}RT}{F}ln\frac{c_{2}}{c_{1}}$$

Equation (30) indicates that the total dissipation of heat produced between the two reference electrodes placed in the system has a contribution of the ohmic resistances of the different parts of the system, but there is also a contribution of the diffusion transport in the solution due to the presence of the boundary diffusion layers, which contribute to the total electric potential drop with both ohmic and non-ohmic phenomena.

We can estimate the total ohmic resistance of the system using the model described in Section 2.2. According to this model, *R_total_* can be expressed as the sum of the total ohmic system resistance in the absence of polarization layers, *R*_0_, and the ohmic resistance change due to the formation of these layers, Δ*R*, expressed by:

(31) $$\Delta R=-\frac{RT}{F\Delta t_{+}I_{L}}+\frac{RT}{IF2\Delta t_{+}}\ln\left(\frac{1+\frac{I}{I_{L}}}{1-\frac{I}{I_{L}}}\right)$$

Using Equations (30) and (31), the total entropy production in the system can be estimated by the following equation:

(32) $${\left.\frac{dS_{i}}{dt}\right|}_{total}=I^{2}\left(\frac{R_{0}}{T}-\frac{R}{I_{L}F\Delta t_{+}}\right)+\frac{IR}{F}\left(\frac{1}{2\Delta t_{+}}+2t_{+}\right)\ln\left(\frac{1+\frac{I}{I_{L}}}{1-\frac{I}{I_{L}}}\right)$$

**Figure 5:**
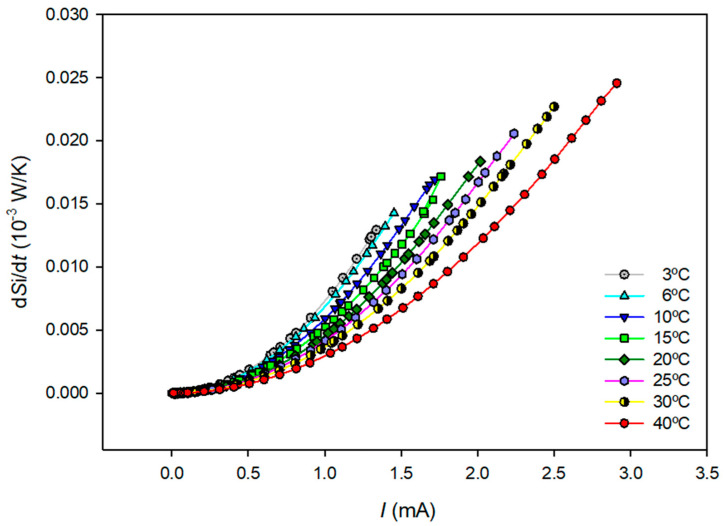
Estimated entropy production as a function of the applied electric current at different temperatures. Lines are provided as visual guides.

Figure 5 shows the values obtained for the total entropy production using Equation (32) and the data in Table 1 as a function of the current density at each temperature.

Visual inspection of Figure 5 shows that the total entropy production increases as the applied electric current increases and decreases as the system temperature increases.

### 4.4. Efficiency Analysis

In the concentration polarization phenomena that occur in electrically driven membrane processes, electric work is partially dissipated as heat due to the irreversible processes occurring in the system. Equation (30) expresses this heat dissipation power, ${\overset{\cdot }{W}}_{\mathrm{dissipated}}$. It is taken from the total electric power input. However, part of the energy is used to create the concentration gradient. Thus, this other part of the power input is converted into chemical energy stored in the saline gradient created in the boundary diffusion layers. In this sense, we can interpret the concentration polarization phenomenon in the system as an energy conversion process, and entropy generation can be used to analyze the energy efficiency of the process. The lower the dissipation, the higher the stored energy. The situation is similar to that in an electrodialysis process, where an electric current is passed through cation and anion exchange membranes placed in alternating order to create a concentration gradient [21].

From the model described in Section 2.2, we can write the electrical power input, $\overset{\cdot }{W}$, as follows:

(33) $$\overset{\cdot }{W}=I\cdot \Delta \varphi_{AB}$$

By combining Equations (3), (32) and (33), the useful electric power is obtained as follows:

(34) $${\overset{\cdot }{W}}_{\mathrm{stored}}=\overset{\cdot }{W}-{\left.T\frac{dS_{i}}{dt}\right|}_{total}=\frac{IRT2{\bar{t}}_{+}}{F}\ln\left(\frac{1+\frac{I}{I_{L}}}{1-\frac{I}{I_{L}}}\right)$$

Equation (34) expresses that the power stored in the salt gradient will be maximum for an ideal selective membrane with ${\bar{t}}_{+}$ = 1. The dissipated power can also be calculated from:

(35) $${\overset{\cdot }{W}}_{\mathrm{dissipated}}={\overset{\cdot }{W}}_{\exp}-{\overset{\cdot }{W}}_{\mathrm{stored}}$$

where ${\overset{\cdot }{W}}_{\exp}$ = *I*Δ*φ* is the experimental power input. In the absence of a membrane, there would be no polarization layers, and the electric power input would be dissipated as Joule heat.

**Figure 6:**
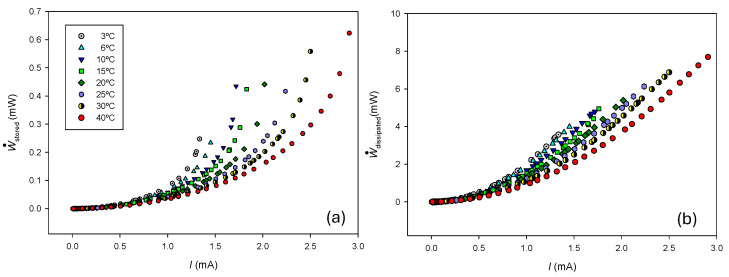
(**a**) Estimated stored and (**b**) dissipated powers as a function of the electric current at different temperatures.

Figure 6 shows the stored and dissipated powers calculated from Equations (34) and (35), experimental current–voltage values, and the data given in Table 1.

As can be observed, both stored and dissipated powers increase with the electric current and decrease with temperature.

System efficiency was evaluated as the store-to-total-power ratio:

(36) $$\eta \left(\%\right)=\frac{{\overset{\cdot }{W}}_{s\mathrm{tored}}}{{\overset{\cdot }{W}}_{\exp}}100$$

**Figure 7:**
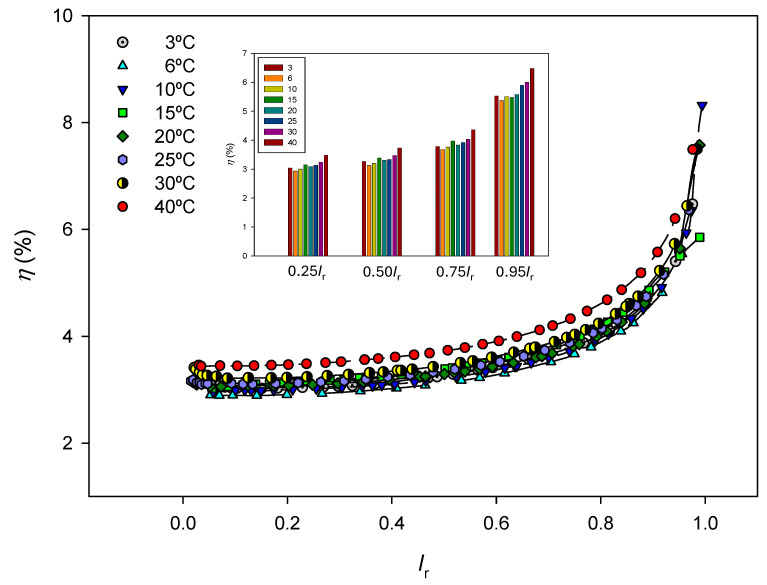
Estimated efficiencies as a function of *I*_r_ = *I*/*I_L_* at different temperatures. Dashed lines are only visual guides. The figure legend shows the efficiency values at different *I*_r_ percentages at the different temperatures for a better visualization.

The efficiency values are shown in Figure 7 as a function of *I*_r_ = *I*/*I_L_* at each temperature. Efficiency increases as the electric current input approaches the corresponding limit value, maybe due to an increase in the concentration difference established between both sides of the membrane. Although it increases both dissipated and stored powers, an increase in temperature generally results in better process efficiency.

It should be noted that the model does not consider the contribution of water transport to entropy production. However, it is expected that this contribution will only be relevant at low flow rates, as the relative importance of the osmotic contribution is reduced.

The model used is only valid for currents below the limiting value, and as the applied current approaches this limiting value, other mechanisms begin to emerge that have not been considered in this study. These mechanisms may contribute to a reduction in the thickness of the boundary diffusion layer and thus may strongly influence the overall entropy production of the system. The analysis of entropy production would be a useful tool to study dissipation effects in the overlimiting regime.

## 5. Conclusions

The entropy production in the polarization phenomena occurring in the underlimiting regime, when an electric current circulates through a single cation-exchange membrane system, has been estimated, and its variation with temperature has been studied.

To this purpose, classical polarization theory and irreversible thermodynamics formalism were used to analyze experimental current–voltage curves of the system at different temperatures.

The results obtained reveal that the total entropy production increases as the applied electric current increases and decreases as the system temperature increases.

The total dissipation of heat in the system has a contribution from the ohmic resistances of the different parts of the system, but there is also a contribution from the diffusion transport in the solution due to the presence of the boundary diffusion layers.

Considering the process as a conversion of electric work input into chemical energy stored in the saline gradient, the system efficiency has been evaluated as the ratio of stored to total power input. The results obtained show that efficiency increases with increasing electric current and temperature.

## Figures and Tables

**Table 1:** Values of the electrical conductivity, equivalent conductance, diffusion coefficient, and transport number for Na^+^ in 5 mol m^−3^ NaCl aqueous solution.

*T* (K)	*κ* (μS cm^−1^) *	*Λ* (Scm^2^mol^−1^) **	*D* (10^−9^ m^2^ s^−1^) **	*t*_+_ **
276.15	350	71.62	0.884	0.360
279.15	376	76.94	0.959	0.386
283.15	415	84.92	1.074	0.389
288.15	474	96.99	1.248	0.392
293.15	535	109.47	1.433	0.394
298.15	608	119.91	1.596	0.395
303.15	654	131.98	1.787	0.397
313.15	753	154.08	2.155	0.399

* Measured. ** Estimated.

**Table 2:** Values of parameters *R*_0_ and y ${\bar{t}}_{+}$, estimated from Equation (3), values of the limiting current estimated from the corresponding Cowan plots, *I_L_* ^Cowan^, and diffusion layer thickness, *δ*, estimated from Peers’ equation.

*T* (K)	*R*_0_ (Ω)	${\bar{t}}_{+}$	*I_L_* ^Cowan^ (10^−3^ A)	*δ* (10^−6^ m)
276.15	1995.6 ± 1.3	0.88 ± 0.10	1.37	545
279.15	1896.17 ± 0.06	0.88 ± 0.06	1.52	555
283.15	1651.6 ± 0.3	0.89 ± 0.08	1.73	540
288.15	1495.00 ± 0.04	0.89 ± 0.03	1.85	592
293.15	1330.8 ± 1.2	0.88 ± 0.15	2.04	633
298.15	1247.0 ± 0.1	0.88 ± 0.08	2.31	629
303.15	1113.9 ± 0.2	0.88 ± 0.06	2.54	635
313.15	921.40 ± 0.04	0.90 ± 0.05	2.98	645

## Data Availability

The data are contained within this article.
